# Medical Electricity

**Published:** 1875-07

**Authors:** 


MEDICAL ELECTRICITY.—Electricity seems to be gaining great favor in the hands of the Medical Profession and as we read the Medical Journals of this and other countries we are pleased to notice the success attending the Profession by the use of this agent in the treatment of so many diseases.

The maladies so treated extend through a wide range, from a little ill to those of a most desperate character.

The use of this important therapeutic agent is fast becoming general and we confess a little surprise at it, for within the past ten years the Physician who used electrical instruments in his practice was pretty sure to be stamped as a Quack, while now an Electro-Magnetic Machine and a Galvanic Battery seem to be almost indispensable to the general practitioner.

We have always claimed it to be the duty of every physician to investigate the reasonableness of new remedies instead of frowning them down as is too frequently the custom, for we need all possible means to alleviate the thousand and one "ills that flesh is heir to."

Our views were happily expressed by Dr. J. M. Tonner, of Washington D. C., President of the American Medical Association, delivered to it at Detroit, June 2, 1874.

"Progress is the order of the day—a law of the universe. He who is not constantly adding to his knowledge and increasing his resources, must soon fall behind the more enterprising and the better informed of his contemporaries. The physician who does not know that the community in which he lives is keeping a constant watch upon him, and contrasting his knowledge, skill, and success in his profession with those of the best and most successful medical men within the range of their reading or acquaintance, shuts his eyes to an important fact of great interest to himself.

---

NOSE-BLEED FROM TIGHT SHOES.—A gentleman of this city told us the other day that his son had suffered greatly from tight or badly formed shoes; he even believed that frequent attacks of bleeding from the nose were caused by the torturing shoes; at least, *the fact* was evident that the young man had nose-bleed when he wore those shoes and did not have it when he changed to a comfortable pair. It may have been a mere coincidence, but the repetition of the occurrence was such as to indicate cause and effect. We were unable to decide as to that; but we were confident in recommending shoes made on the McComber system that we were prescribing a sure remedy for much of the suffering which the young man experienced.

